# The contributions of muscarinic receptors and changes in plasma aldosterone levels to the anti-hypertensive effect of *Tulbaghia violacea*

**DOI:** 10.1186/1472-6882-13-13

**Published:** 2013-01-11

**Authors:** Ismaila Raji, Pierre Mugabo, Kenechukwu Obikeze

**Affiliations:** 1 Faculty of Medicine, National University of Science and Technology, P.O. Box AC 939 Ascot, Bulawayo, Zimbabwe; 2 School of Pharmacy, University of the Western Cape, Private Bag X17, Cape Town, 7535, South Africa

**Keywords:** *Tulbaghia violacea*, Spontaneously hypertensive rats, Blood pressure, Heart rate, Muscarinic receptors, Aldosterone

## Abstract

**Background:**

*Tulbaghia violacea* Harv. (Alliaceae) is used to treat various ailments, including hypertension (HTN) in South Africa. This study aims to evaluate the contributions of muscarinic receptors and changes in plasma aldosterone levels to its anti-hypertensive effect.

**Methods:**

In the acute experiments, methanol leaf extracts (MLE) of *T. violacea* (30–120 mg/kg), muscarine (0.16 -10 μg/kg), and atropine (0.02 - 20.48 mg/kg), and/or the vehicle (dimethylsulfoxide (DMSO) and normal saline (NS)) were respectively and randomly administered intravenously in a group of spontaneously hypertensive (SHR) weighing 300 to 350 g and aged less than 5 months. Subsequently, *T. violacea* (60 mg/kg) or muscarine (2.5 μg/kg) was infused into eight SHRs, 20 min after atropine (5.12 mg/kg) pre-treatment. In the chronic (21 days) experiments, the SHRs were randomly divided into three groups, and given the vehicle (0.2 ml/day of DMSO and NS), *T. violacea* (60 mg/kg/day) and captopril (10 mg/kg/day) respectively into the peritoneum, to investigate their effects on blood pressure (BP), heart rate (HR), and plasma aldosterone levels. Systolic BP and HR were measured using tail-cuff plethysmography during the intervention. BP and HR were measured via a pressure transducer connecting the femoral artery and the Powerlab at the end of each intervention in the acute experiment; and on day 22 in the chronic experiment.

**Results:**

In the acute experiments, *T. violacea,* muscarine, and atropine significantly (p < 0.05) reduced BP dose-dependently. *T. violacea* and muscarine produced dose-dependent decreases in HR, while the effect of atropine on HR varied. After atropine pre-treatment, dose-dependent increases in BP and HR were observed with *T. violacea*; while the BP and HR effects of muscarine were nullified. In the chronic experiments, the *T. violacea*-treated and captropril-treated groups had signicantly lower levels of aldosterone in plasma when compared to vehicle-treated group. Compared to the vehicle-treated group, significant reduction in BP was only seen in the captopril-treated group; while no difference in HR was observed among the groups.

**Conclusion:**

The results obtained in this study suggest that stimulation of the muscarinic receptors and a reduction in plasma aldosterone levels contribute to the anti-hypertesive effect of *T. violacea*.

## Background

Globally, sixty per cent of the burden of cardiovascular disease (CVD) and about fifty percent of that of coronary heart disease (CHD) is due to hypertension (HTN). Furthermore, in developing countries, a rapid increase in CVD which mirrors the global trend of rapid urbanization is seen [1], and the overall burden of CVD has been predicted to rise by approximately 150% in these countries in the next 20 years [2]. This may be partly due to a decline in deaths in infancy, childhood, and adolescence from infections [1]. In Africa, CVD affects 1.3 million people yearly [3,4]; and Africans [5] and African Americans [6,7] respond with heightened vascular reactivity when exposed to stress. The prevention and treatment of risk factors for CVD are effective and sustainable, but poverty and cultural factors hinder the implementation of the DASH (Dietary Approaches to Stop Hypertension) [1,8,9] found to be very effective in African-Americans [10]. Furthermore, developing countries, particularly those in sub-Saharan Africa are financially unable to intervene in all the risk factors for CVD [8,9], necessitating a continuous search for better and cheaper alternate methods for controlling blood pressure (BP) [11].

Africa has a high rate of endemism of medicinal plants [12,13] which are used by rural communities to treat various ailments, including HTN [14-17]. *Tulbaghia violacea* (*T. violacea*), an example of these plants [18-20] has attracted a lot of interest in the scientific community of late [20-23]. In the field of cardiovascular research, *T. violacea* has been reported to reduce blood pressure in spontaneously hypertensive rats (SHR) [21], normotensive Wistar rats [23] and Dahl salt sensitive (DSS) rats [24], which were associated with inhibition of the β_1_ adrenoceptors [21], inhibition of the angiotensin I converting enzyme (ACE) [21,23], and decreased renal angiotensin II type 1 (AT_1_) receptor gene expression [24]. Raji, Mugabo *et al*., [21] also reported a reduction in heart rate (HR) with *T. violacea*. Consequently, the present study will investigate possible contributions of muscarinic receptors to the acute anti-hypertensive effect of *T. violacea*, and also investigate the effect of chronic administration of *T. violacea* or captopril on BP, HR, plasma aldosterone levels, and body weight in the SHR.

## Methods

### Plant material

Fresh plants were purchased from the New Plant Nursery, George, South Africa in August and September, 2008; identified by the taxonomist at the Department of Biodiversity and Conservation Biology of the University of the Western Cape (UWC), Bellville; and deposited at the herbarium with voucher numbers 6955 and 6956.

### Plant extraction

Fresh leaves weighing 2.4 kg were washed, and dried in an oven at 30°C until a constant mass was obtained. Dried leaves were ground into powder. Methanol leaf extract (MLE) was prepared by Soxhlet extraction and the excess solvent removed at 40°C using a rotavapor. The dried black paste obtained was placed in a −20°C freezer before being dried further using a freeze-drier. The final dried extract (76.6 g or 3.2% yield) was stored in a brown bottle in a refrigerator at −4°C. Fresh MLE was dissolved with drops of dimethylsulfoxide (DMSO) and the required concentration made up with 0.9% normal saline and filtered before infusion into the rat to prevent the formation of emboli.

### Animals

Healthy male SHR weighing 300–350 g, aged less than 5 months old were obtained from the Animal Unit at the University of Cape Town, South Africa; and were kept under laboratory conditions in the animal room, School of Pharmacy, UWC; and given water and feed *ad libitum*. Room temperature was kept at 24°C, with a 12:12-h light–dark cycle.

### Drugs

Atropine (atropine sulfate salt monohydrate), muscarine ((+)-muscarine chloride) and captopril were purchased from Sigma-Aldrich, South Africa. Solvents were 0.9**%** saline (Adcock Ingram, South Africa) and dimethylsulfoxide (DMSO, Merck Chemicals, South Africa). Atropine and muscarine were dissolved in 0.9**%** saline, while *T. violacea* and captopril were initially dissolved with drops of DMSO, and made up to the required dilution (concentration) using 0.9**%** saline. Fresh drug solutions were prepared at the beginning of each experiment and kept on ice during the course of the experiment.

### *In- vivo* experiments

Rats were anaesthetized with 6% sodium pentobarbitone (Kyron Laboratories, South Africa) at a dose of 40 mg/kg intraperitoneally, and fastened in a supine position on a heated rat-operating table (BioScience), whose temperature was maintained at 37.3 ± 0.5°C by a thermostat. A temperature probe (AD Instruments) was inserted into the rectum to monitor the body temperature. The trachea was cannulated to maintain airflow during the experiment and an oxygen mask placed close to the opening of the tracheal cannula to maintain adequate supply of oxygen (Afrox, South Africa) to the rat. The right external jugular vein was cannulated with a small polyethylene cannula to allow intravenous infusion of drugs via a syringe placed on the two-way injection Ascor AP 22 syringe pump (Poland). The left femoral artery was cannulated with a small polyethylene cannula filled with heparin (Intramed, South Africa) 100 IU/ml normal saline [25]. The femoral cannula was connected to a BP transducer attached to a BP amplifier (ADInstruments, Australia) and Power Lab 4/20 T (ADInstruments, Australia) for recordings of the BP and HR onto a Chart 5.0 for Windows software (ADInstruments, Australia). Rats were allowed a 15-min stabilization period to ensure that BP and HR parameters were stable before any further experiment, and each group consisted of 8 rats. Drugs were infused, flushed with 0.1 ml of normal saline and results recorded within 3 min of infusion. Pressures and HR were allowed to return to baseline values (10–15 min) before further doses were infused.

### Tail-cuff plethysmography

In conscious SHR, systolic BP was measured using non-invasive tail-cuff plethysmography (ADInstruments, Australia), between 09:00 and 16:00 h. The SHRs were warmed at 37°C for 10 min and allowed to rest in a glass restrainer, and a black conical plastic piece with a nose opening, placed over the head region of the rat, to cover the eyes of the rat, and consequently allow the rat to rest better; before the tail-cuff plethysmography. An average of 5 consecutive readings was recorded. Rats were trained every 3 days for BP measurement for two weeks prior to the start of the actual experiment [26-29].

### Experimental protocol

a) Muscarinic receptors

Dose–response experiments (DRE) for muscarine (0.16 -10 μg/kg) and atropine (0.02 - 20.48 mg/kg) were performed in the SHR. The dose at which 80% of the maximum effect obtained was noted.

Previously [21], 80% of the maximum effect of *T. violacea*, was achieved at 60 mg/kg. Therefore, this dose (60 mg/kg), half of it (30 mg/kg), and twice this dose (120 mg/kg) were used in this study. *T. violacea* (30, 60 and 120 mg/kg) was infused into eight SHRs with the BP and HR values allowed to return to baseline, prior to the administration of the subsequent dose. Another set of eight SHRs were pre-treated with atropine (5.12 mg/kg). In this study, *T. violacea* or muscarine was infused 20 min [30-33] after atropine was infused into rats. *T. violacea* (30, 60 and 120 mg/kg) was then infused, with the BP and HR values allowed to return to baseline, prior to the subsequent dose of the MLE being administered.

b) *T. violacea* effect compared with muscarine chloride after atropine pre-treatment.

Eight SHRs were pre-treated with atropine (5.12 mg/kg), before *T. violacea* (60 mg/kg) was infused into these rats. The BP and HR returned to baseline, before muscarine (2.5 μg/kg) was infused into the same rat.

The effects of the MLE and the standard drugs on SBP, DBP, MAP and HR were evaluated.

c) Body weight, BP, HR and plasma aldosterone levels

The SHRs used in this protocol were allowed 14 days of acclimatization to the restrainers [26-29]. The body weight, SBP and HR of the rats were measured on the first day of study, and the rats were randomly divided into three groups of eight each. The intraperitoneal injection given to the rats during the next 21 days were *T. violacea* (60 mg/kg), captopril (10 mg/kg) or 0.2 mls of the vehicle (DMSO + NS). The BP and HR of the rats were measured using the non-invasive tail cuff plethysmography during the intervention period. On day 22, invasive BP measurement was done, and blood was collected from the femoral artery of the SHR after the BP and HR values had stabilized and recorded. Blood was stored in ethylenediaminetetraacetic acid (EDTA) tubes and rapidly spun at 10 000 rpm, in a centrifuge to separate the plasma from the blood cells. The plasma obtained was then stored in another set of EDTA tubes and placed in a – 40°C freezer. Plasma samples were sent to the Veterinary Hormone Laboratory, Faculty of Veterinary Science, University of Pretoria (South Africa), for analysis of plasma aldosterone levels.

### Data analysis

The paired and/or unpaired Student’s T test was used to calculate statistical significance (p < 0.05), using the Microsoft Excel software.

### Ethical considerations

The methodology and ethics adhered to in this study were approved by the Ethics Committee of the University of the Western Cape, and the registration number obtained was 09/7/35. All experimental procedures used in this study were conducted in accordance with the guidelines provided by the European Community guidelines (EEC Directive of 1986; 86/609/EEC).

## Results

### Effect of (+)-muscarine chloride on BP and HR in SHR

**Figure 1:**
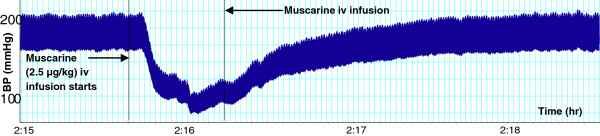
Effect of muscarine (2.5 µg/kg) on BP in a SHR. Chart scaling 100:1.

**Figure 2:**
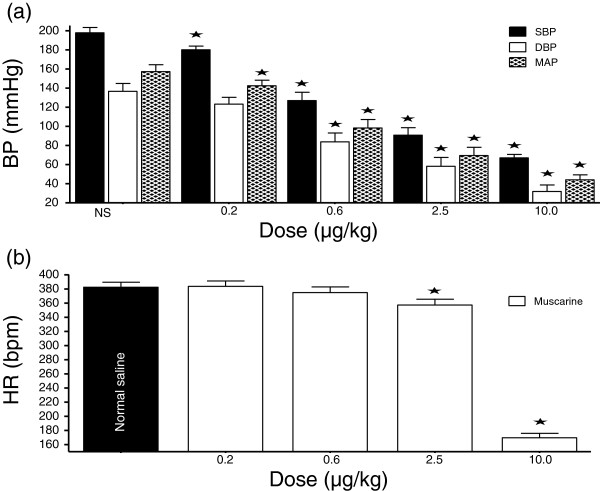
**Dose-response graph of the effect of muscarine (0.2 – 10.0 µg/kg) on BP and HR. **Values are presented as mean ± SEM. * Indicates statistical significance.

In a dose-dependent fashion, muscarine (0.2 – 10.0 μg/kg) significantly reduced the SBP, DBP and MAP. The reductions in HR were only significant at the two highest doses (Figures 1 and 2).

### Effect of atropine on BP and HR in SHR

**Figure 3:**
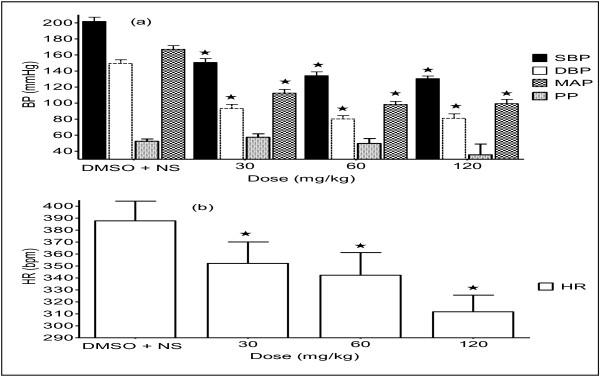
**Effect of atropine (5.12 mg/kg) on BP in a SHR. **Chart scaling 100:1.

**Figure 4:**
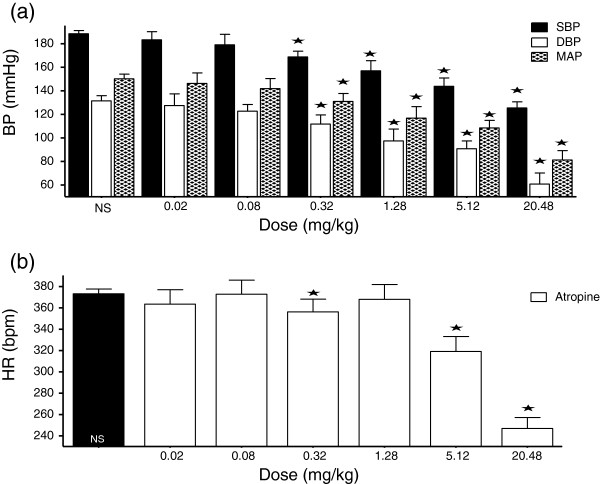
**Dose-response graph of the effect of atropine (0.02 – 20.48 mg/kg) on BP and HR. **Values are presented as mean ± SEM. * Indicates statistical significance.

In a dose-dependent fashion, atropine (0.02 - 20.48 mg/kg) significantly reduced the SBP, DBP, and MAP. The changes in HR with atropine were not dose-dependent (Figures 3 and 4).

### Effect of *T. violacea* on BP and HR

*T. violacea* (30, 60 and 120 mg/kg) significantly reduced the SBP, DBP, MAP and HR in a dose-dependent manner (Figure 3).

### Effect of *T. violacea* on BP and HR, after pre-treatment with atropine

**Figure 5:**
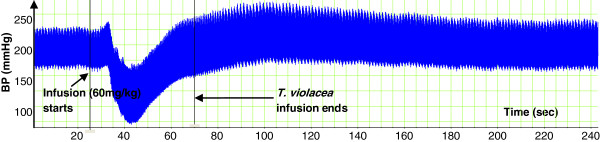
**Effect of T. violacea (60 mg/kg) on BP in a SHR. **Chart scaling 100:1.

**Figure 6:**
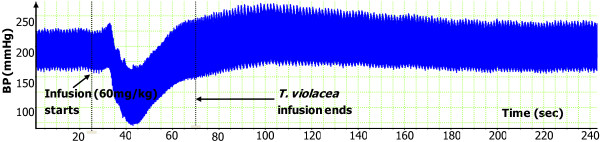
**Effect of T. violacea (30, 60 and 120 mg/kg) on BP (a) and HR (b). PP is pulse pressure. **Values are presented as mean ± SEM. * Indicates statistical significance.

**Figure 7:**
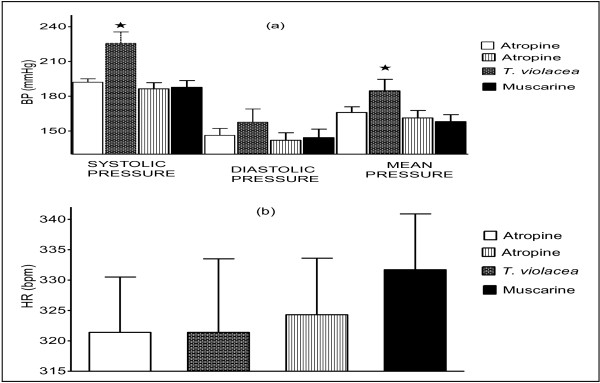
**Effect of T. violacea (60 mg/kg) on BP, after atropine pre-treatment. **Chart scaling 100:1.

**Figure 8:**
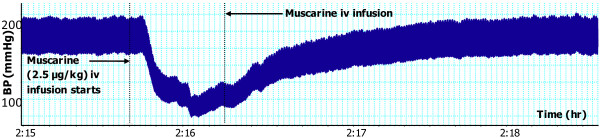
**Effect of T. violacea (30, 60 and 120 mg/kg) on BP (a) and HR (b) after atropine pre-treatment. **PP is pulse pressure. Values are presented as mean ± SEM. * Indicates statistical significance.

Simultaneous co-infusion of atropine (5.12 mg/kg) with *T. violacea* (60 mg/kg) produced BP changes in the rats similar to those obtained with the infusion of the MLE alone (results not shown here). However, the infusion *T. violacea*, 20 min after pre-treatment of the rats with atropine (5.12 mg/kg), led to dose-dependent increases in the BP and HR of the rats (Figures 7 and 8), as opposed to the hypotensive effect previously seen with the infusion of doses of *T. violacea* alone (Figures 5 and 6). The BP increases were significant at all doses, but the HR increases was only significant at the highest dose (Figure 8).

### Comparing *T. violacea* with (+)-muscarine chloride

**Figure 9:**
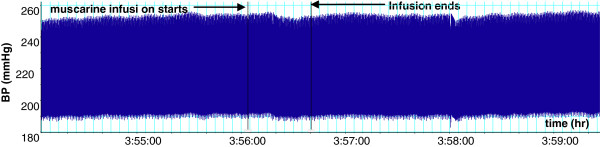
**Effect of muscarine (2.5 μg/kg) on BP after atropine pre-treatment. **Chart scaling 100:1.

**Figure 10:**
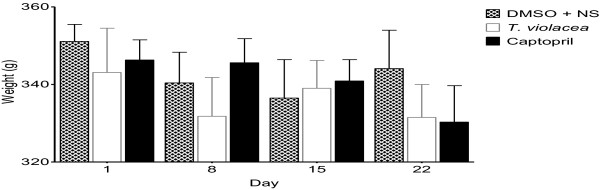
**Effect on BP (a) and HR (b) of T. violacea (60 mg/kg) or muscarine (2.5 µg/kg) after atropine pre-treatment. **Values are presented as mean ± SEM. * Indicates statistical significance.

As, above, the infusion of *T. violacea* (60 mg/kg) in rats pre-treated with atropine (5.12 mg/kg) produced significant increases in SBP and MAP (Figure 10), but the BP and HR effects of muscarine (2.5 μg/kg) (Figures 1 and 2) were nullified (Figures 9 and 10).

### Effect of chronic administration of the vehicle, *T. violacea* or captopril on body weight

**Figure 11:**
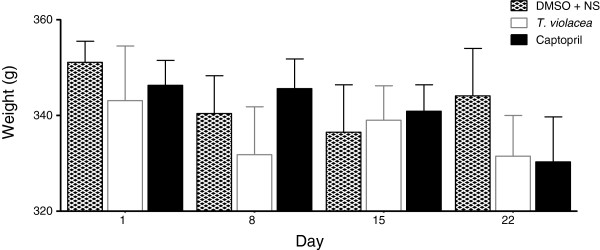
**Effect of chronic administration of the vehicle, T. violacea or captopril on body weight. **Values are presented as mean ± SEM. * Indicates statistical significance.

Intraperitoneal administration of the vehicle (DMSO + NS), *T. violacea* (60 mg/kg/day) or captopril (10 mg/kg/day) for 21 days did not produce any significant change in the mean body weight of any of the SHR groups (Figure 11).

### Effect of chronic administration of the vehicle, *T. violacea* or captopril on blood pressure

**Figure 12:**
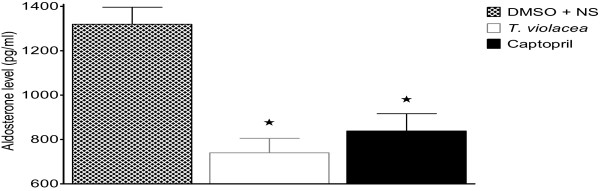
**Effect of chronic administration of the vehicle, T. violacea or captopril on BP (a) and HR (b) of male SHR. **Values are presented as mean ± SEM. * Indicates statistical significance.

The SBP values obtained using the invasive BP measure technique were significantly lower on day 22 when compared to the values obtained using the non-invasive tail cuff technique on day 1, in all groups. These reductions in SBP were most significant in the captopril group, while the reduction in SBP in *T. violacea* group was similar to that of the control group. Significant reductions in HR also occurred in the control and captopril groups (result not shown here). After 21 days, significant lower SBP, DBP or MAP values were only seen in the captopril group when compared with the control group. There was no significant difference in final HR among the groups (Figure 12).

### Effect of chronic administration of the vehicle, *T. violacea* or captopril on plasma aldosterone level

**Figure 13:**
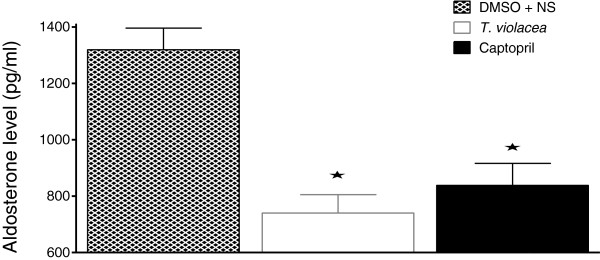
**Effect of chronic administration of the vehicle, T. violacea or captopril on plasma aldosterone levels. **Values are presented as mean ± SEM. * indicates statistical significance.

Compared to the plasma aldosterone levels (1319.5 ± 76.7 pg/ml) in the control group significantly lower values of 740.2 ± 65.0 pg/ml and 838.4 ± 77.9 pg/ml were observed in the *T. violacea* and captopril treated groups respectively (Figure 13).

## Discussion

### Muscarinic receptors

In the present study, both muscarine and atropine produced reductions in BP (Figures 1,2,3 and 4). While muscarine produced reductions in HR (Figure 2), the effect of atropine on HR varied (Figure 4). Lower doses of atropine did not produce any appreciable change in BP or HR, as opposed to some reports in literature [34,35]. In literature, muscarine chloride has been reported to have similar activity as acetyl choline, such as reducing BP and HR; although being more potent [36]. Muscarine reduces BP, while atropine has various effects on BP, from increases [36-38], to no effect in normotensive Wistar Kyoto (WKY) rats and SHR [39,40], and decreases at doses of 5–50 mg/kg intravenously infused to conscious un-anaesthetized WKY rats and hypertensive rats (SHR, and Sprague–Dawley rats made hypertensive by subcutaneous implantation of deoxycorticosterone acetate) [41-43]. Atropine has also been reported to produce vasodilation [28,44].

In rats pre-treated with atropine, the infusion of the *T. violacea* (30 mg, 60 mg and 120 mg/kg), led to significant dose-dependent increases in BP (Figures 7 and 8), as opposed to dose dependent decreases in the absence of atropine (Figures 5 and 6). Dose-dependent increases in HR were also observed, which was only significant at the highest dose given (Figure 8), as opposed to significant dose-dependent decreases in HR observed in the absence of atropine (Figure 3). This suggests that the MLE contains compounds, which act through the muscarinic receptors. It may also suggest that the MLE, being crude, also contains some substances, which may have BP increasing effect, which become pronounced when they act on other receptors in the absence of the action of the MLE on the muscarinic receptors. Interestingly, a large proportion of patients with HTN have increased sympathetic activity, associated with decreased parasympathetic activity [45,46].

In some experiments, the effect of co-infusing atropine with *T. violacea* was tested. The BP profile and BP reductions observed were similar to those seen in the absence of atropine (results not shown here), which suggests that atropine takes some time before it adequately blocks the muscarinic receptors. Consequently, the infusion of atropine in this study was done 20 min or more before either muscarine or *T. violacea* was infused into the same animal, as was done by previous researchers who tested blockade of muscarinic receptors in other plants and drugs [30-33]. In experiments that compared the effect on BP and HR of infusing muscarine (2.5 μg/kg) (known agonist of the muscarinic receptors) [36], or *T. violacea* (60 mg/kg) in rats pre-treated with atropine; the effect of muscarine on BP and HR (Figures 1 and 2) were nullified (Figures 9 and 10), while *T. violacea* increased SBP and MAP (Figure 10). This experiment further suggests that activation of the muscarinic receptors in the heart, and consequent bradycardia [47] contributes to the hypotensive effects of both muscarine and *T. violacea.*

### Plasma aldosterone levels

Chronic administration of the vehicle (DMSO + NS), *T. violacea* (60 mg/kg/day) or captopril (10 mg/kg/day) did not produce any significant change in the mean body weight of any of the SHR groups (Figure 10). The SBP values obtained using the invasive BP measure technique were significantly lower on day 22 when compared to the values obtained using the non-invasive tail cuff technique on day 1, in all groups. These reductions in SBP were most significant in the captopril group, while the reduction in SBP in *T. violacea* group was similar to that of the control group. Significant reductions in HR also occurred in the control and captopril groups (result not shown here). After 21 days, significantly lower SBP, DBP or MAP values were only seen in the captopril group when compared with the control group. There was no significant difference in final HR among the groups (Figure 11). The effect of chronic captopril administration on BP is in line with literature. Captopril is an angiotensin converting enzyme (ACE) inhibitor, which reduces BP [48-50]. Reports in literature show that acute captopril administration may decrease [51,52], decrease or have no effect [53] on HR, while chronic captopril administration may decrease [54] or increase HR [48]. The higher BP and HR readings observed with the tail-cuff measurements in the conscious rats earlier in the study, when compared to the values obtained in anaesthetized rats with the invasive BP measurement, may be partly due to sympathetic activation [29,55,56]. This may be secondary to mental stress in the conscious rats, despite the initial two weeks of acclimatization given to all rats used in the study. Secondly non-invasive BP measurements are not very reliable [29]. These results suggest that chronic administration of *T. violacea* may not reduce BP or HR in the SHR. This is contrary to the reported reduction in BP in the DSS rats by Mackraj, Ramesar *et al.*, [24].

The significantly lower plasma levels of aldosterone in the *T. violacea* group, when compared with the control group (Figure 13) agrees with Mackraj, Ramesar *et al.,*[24]. However, the significantly lower plasma concentration in the captopril group when compared to the control group seen in this study is different from that observed by Mackraj, Ramesar *et al.,*[24], who did not observe significantly lower plasma aldosterone level in the captopril group when compared to the control group in their study (Figure 13). Aldosterone regulates electrolyte, fluid balance and BP homoeostasis [57]. It also mediates maladaptive tissue remodelling throughout the CVS and central nervous system (CNS), thereby, worsening the HTN [58-60]. Therefore, a reduction in its plasma levels is advantageous to the rat.

## Conclusion

The results of this study show that the antihypertensive effects of the crude methanol leaf extract of *T. violacea* in male SHR may involve the stimulation of muscarinic receptors, and reduction in plasma aldosterone levels. Further studies are required to separate the chemical compounds present in *T. violacea* and determine the pharmacological actions of each of them.

## Abbreviations

BP: Blood pressure, bpm, Beats per minute; CHD: Coronary heart disease; CVD: Cardiovascular disease; DBP: Diastolic blood pressure; DMSO: Dimethylsulfoxide; DRE: Dose- response experiment; DSS: Dahl salt sensitive; EDTA: Ethylenediaminetetraacetic acid; HR: Heart rate; iv: Intravenous; HTN: Hypertension; MAP: Mean arterial pressure; MLE: Methanol leaf extract; mmHg: Millimetres of mercury; NS: Normal saline; PP: Pulse pressure; SBP: Systolic blood pressure; SEM: Standard error of mean; SHR: Spontaneously hypertensive rats; *T. violacea*: *Tulbaghia violacea*; UWC: University of the Western Cape; WKY: Wistar-Kyoto rats.

## Competing interests

The authors declare that they have no competing interests.

## Authors’ contributions

PM conceived the study and participated in the design, acquisition, analysis, and interpretation of data. KO participated in the conceptualization of the design, acquisition, analysis, and interpretation of data. IR participated in the conceptualization of the design, acquisition, analysis, and interpretation of data, and carried out the technical aspect of the study. All authors read and approved the final manuscript.

## Authors’ information

IR has a PhD in Pharmaceutical Sciences, M.Sc and B.Sc Honours degrees in Human Physiology. IR is currently a Senior Lecturer in Physiology, at the National University of Science and Technology, Bulawayo, Zimbabwe.

## Pre-publication history

The pre-publication history for this paper can be accessed here:

http://www.biomedcentral.com/1472-6882/13/13/prepub
